# Supplementary material for: Food Insecurity and Water Insecurity in Rural Zimbabwe: Development of Multidimensional Household Measures
Source: Int J Environ Res Public Health. 2021 Jun 3;18(11):6020. doi: 10.3390/ijerph18116020 (PMC8199942; doi:10.3390/ijerph18116020)
Supplement: Supplementary file 1 [file ijerph-18-06020-s001.zip › ijerph-1207832-supplementary.pdf]

Table S1. Description of missing item variables (N=4675).

	Variable	N Missing	% missing
Food	Garden	19	0.41
	Stock of staple food	57	1.22
	Food not prefered	13	0.28
	Insufficient food	25	0.53
	Food help	46	0.98
	Assets sold for food	18	0.39
	Food on credit	51	1.09
	Time to food market	52	1.11
	Handwashing	47	1.01
	Food storage location	844	18.05
	Household diet diversity	25	0.53
	Social shocks	10	0.21
	Economic shocks	58	1.24
	Agriculture shocks	10	0.21
	Health shocks	10	0.21
Water	Water volume	970	20.75
	Time to drinking source	0	0
	Distance to drinking source	68	1.45
	Time to non-drinking source	0	0
	Distance to non-drinking source	80	1.71
	Drinking source	64	1.37
	Water container	39	0.83
	Water satisfaction	409	8.75
	Water treatment	129	2.76
	Non-drinking source	83	1.78
	Frequency non-drinking water source ran dry	100	2.14
	Frequency drinking water source ran dry	78	1.67

Table S2. Comparison of baseline characteristics of participants with complete vs. missing values.

Characteristics*	Food insecurity sample			Water insecurity sample		
	Complete (N=3551)	Missing (N=1071)	p**	Complete (N=3311)	Missing (N=1264)	p**
**SES status**			<0.01			0.09
Lower tertile	1141 (32.17)	410 (38.35)		1079 (32.63)	452 (35.93)	
Middle tertile	1192 (33.61)	338 (31.62)		1117 (33.78)	393 (31.24)	
Upper tertile	1214 (34.23)	321 (30.03)		1111 (33.6)	413 (32.83)	
**Women living with HIV**			0.01			0.10
Unknown	14 (0.39)	8 (0.75)		14 (0.42)	8 (0.63)	
Yes	556 (15.66)	204 (19.05)		524 (15.83)	230 (18.2)	
No	2981 (83.95)	859 (80.21)		2773 (83.75)	1026 (81.17)	
**Relationship status**			0.85			0.86
Not partnered	151 (4.46)	47 (4.6)		141 (4.47)	55 (4.59)	
Partnered	3233 (95.54)	975 (95.4)		3015 (95.53)	1142 (95.41)	
**Women's education level**			0.23			0.17
Primary	640 (18.75)	217 (21.09)		596 (18.76)	249 (20.54)	
Some secondary	1263 (37.01)	376 (36.54)		1199 (37.74)	423 (34.9)	
Completed secondary	1510 (44.24)	436 (42.37)		1382 (43.5)	540 (44.55)	
**Religion**			0.69			0.05
Other	1815 (53.21)	556 (53.93)		1663 (52.36)	675 (55.6)	
Apostolic	1596 (46.79)	475 (46.07)		1513 (47.64)	539 (44.4)	
**Women's employment**			0.18			0.51
Not employed	3241 (91.58)	963 (90.25)		3018 (91.4)	1143 (90.79)	
Employed	298 (8.42)	104 (9.75)		284 (8.6)	116 (9.21)	
**Women's depression**			<0.01			0.01
Depressed	3284 (94.23)	961 (91.61)		3061 (94.13)	1132 (91.88)	
Not depressed	201 (5.77)	88 (8.39)		191 (5.87)	100 (8.12)	
**Nulliparous**			0.88			0.56
No	1999 (84.42)	568 (84.65)		1964 (84.29)	576 (85.21)	
Yes	369 (15.58)	103 (15.35)		366 (15.71)	100 (14.79)	
**Women's age, n/ mean (SD)**	3401/ 26.42 (6.72)	1024/ 25.79 (6.78)	0.01	3171/ 26.32 (6.68)	1207/ 26.05 (6.86)	0.23
**Perceived Health Status, n/ mean (SD)**	3065/ 3.42 (0.99)	929/ 3.34 (1.03)	0.05	2874/ 3.41 (0.99)	1078/ 3.57 (1.54)	0.21
**Perceived Time Stress, n/ mean (SD)**	3473/ 2.65 (0.71)	1028/ 2.68 (0.73)	0.32	3239/ 2.66 (0.72)	1215/ 2.65 (0.71)	0.70
**Household size, n/ median (IQR)**	3432/ 5 (3)	1040/ 5 (3)	0.18	3199/ 5 (3)	1221/ 4 (3)	<0.01
**Season**			0.05			<0.01
Rainy: November to March	1604 (45.25)	521 (48.74)		1473 (44.5)	653 (51.83)	
Dry: April to October	1941 (54.75)	548 (51.26)		1837 (55.5)	607 (48.17)	
**Season**			0.01			<0.01
Hungry: January to March	1030 (29.06)	356 (33.3)		942 (28.46)	447 (35.48)	
Plenty: April to December	2515 (70.94)	713 (66.7)		2368 (71.54)	813 (64.52)	
**Calendar quarter**			0.04			<0.01
January to March	1030 (29.06)	356 (33.3)		942 (28.46)	447 (35.48)	
April to June	780 (22)	225 (21.05)		756 (22.84)	221 (17.54)	
July to September	835 (23.55)	221 (20.67)		768 (23.2)	274 (21.75)	
October to December	900 (25.39)	267 (24.98)		844 (25.5)	318 (25.24)	

* All characteristics presented as n (%) unless otherwise specified

** Statistically significant difference (p<0.05) between those with complete vs those with missing variables

Table S3. Correlation (rho) between the scores of food insecurity dimensions and water insecurity dimensions

Dimensions	Poor food access	Household shocks	Low food quality and availability	Poor water access	Poor water quality	Low water reliability
Poor food access	1.00					
Household shocks	0.01	1.00				
Low food quality and availability	0.02	0.04	1.00			
Poor water access	**0.07**	**0.05**	**0.07**	1.00		
Poor water quality	**0.12**	0.02	**0.05**	-0.03	1.00	
Low water reliability	**0.07**	**0.09**	-0.04	0.00	0.01	1.00

Bolded vaues indicate correlation coefficients significant at $p<0.05$

Table S4. Sensitivity analysis of multiple correspondence analyses with imputed variables

Food insecurity (N=4,622)			
**MCA food dimensions**	**1**	**2**	**3**
**Dimension names**	**Poor food access**	**Household shocks**	**Low food availability and quality**
**Stock of staple food**	0.08	0.03	**0.30**
**Garden**	0	0	**0.27**
**Food not preferred**	**0.50**	0.01	0.0
**Insufficient food**	**0.53**	0	0.02
**Food help**	**0.48**	0	0
**Food on credit**	**0.50**	0.00	0
Assets sold for food	0.03	0.03	0.02
Time to food market	0.00	0.01	0.01
Handwashing	0	0.03	0.09
Food storage location	0.02	0	0.06
**Household diet diversity**	0.01	0.02	**0.38**
Social shocks	0.01	0.08	0.00
**Economic shocks**	0.01	**0.35**	0.00
**Agriculture shocks**	0.01	**0.40**	0
**Health shocks**	0	**0.41**	0
**Eigenvalue**	2.19	1.42	1.28
**% variance**	9.13	5.90	5.35

Water insecurity (N=4,575)			
**MCA water dimensions**	**1**	**2**	**3**
**Dimension names**	**Poor water access**	**Poor water quality**	**Low water reliability**
Water volume	0.00	0.01	0.01
**Time to drinking source**	**0.56**	0.00	0.00
**Distance to drinking source**	**0.49**	0.00	0.00
**Time to non-drinking source**	**0.57**	0.00	0.00
**Distance to non-drinking source**	**0.50**	0.00	0.00
**Drinking source**	0.11	**0.77**	0.07
**Non-drinking source**	0.11	**0.66**	0.02
**Water satisfaction**	0.04	**0.29**	0.01
Water treatment	0.00	0.04	0.00
Water container	0.00	0.00	0.01
**Frequency of availability of drinking water at the source**	0.00	0.00	**0.70**
**Frequency of availability of non-drinking water at the source**	0.00	0.00	**0.69**
**Eigenvalue**	2.37	1.77	1.51
**% variance**	13.18	9.85	8.38

Item variables in bold were identified as relevant and further indicate which dimension they load on.
